# Knowledge of Fertile Period and Its Determinants Among Women of Childbearing age in Ethiopia: A Multilevel Analysis Based on 2016 Ethiopian Demographic and Health Survey

**DOI:** 10.3389/fpubh.2022.828967

**Published:** 2022-05-19

**Authors:** Maereg Wolde, Ayenew Kassie, Kegnie Shitu, Zelalem Nigussie Azene

**Affiliations:** ^1^Department of Health Education and Behavioral Sciences, College of Medicine and Health Science, University of Gondar, Gondar, Ethiopia; ^2^Department of Women's and Family Health, School of Midwifery, College of Medicine and Health Sciences, University of Gondar, Gondar, Ethiopia

**Keywords:** fertile period, knowledge, determinant factors, multilevel, EDHS background

## Abstract

**Background:**

The knowledge of the fertile period is one of the science techniques used to delay pregnancy. Although it is a highly effective method, most women lack correct knowledge about it and end up with unintended pregnancies and undergo through unsafe abortion, which is among the leading factors for maternal death. Therefore, this study is aimed to assess the knowledge about fertile period and its determinant factors among reproductive age women in Ethiopia.

**Methods:**

The data were extracted from the 2016 national cross-sectional Ethiopian Demographic and Health Survey. The data were collected using a two-stage cluster design. Descriptive statistics were used to summarize the study findings. The determinants of knowledge about fertile period were analyzed using a multilevel binary logistic regression model.

**Results:**

A total of 15,683 women were included. From this, 23.6% (95% *CI*: 23–24) had knowledge about fertile period. Age group of 20–24 years, 25–29 years, 30–34 years, 35–39 years, 40–44 years, and 45–49 years; accomplishment of primary education, secondary education, and higher education; partner high level of education; wealth status of poorer, middle, richer, and richest; a person listened to radio < once a week; and a person who watch TV at least once in a week and who ever heard about family planning; internet usage in the last 12 months, being protestant religion follower; and community family planning message exposure were significantly associated with knowledge about fertile period.

**Conclusion:**

The number of reproductive age women who know about fertile period is low in Ethiopia. Age above 19 years, respondent's education attainment from primary to higher education, partner high level of education, being from poorer to richest wealth status, listening to radio, watching TV, ever heard of FP, internet usage in the last 12 months, being protestant religion follower, and community family planning exposure were significantly associated with good knowledge about fertile period.

## Introduction

The knowledge regarding fertile period is one of the natural family planning methods, which apply scientific techniques used to postpone pregnancy. It is mainly used by those who don't want to use mechanical, hormonal, or surgical means of contraception (1). This method includes the basal body temperature method, the cervical mucus (or Billings) method, and the symptom thermal method. These three methods are helpful to determine the fertile period and for its successful application. For periodic abstinence, the women need to correctly identify the fertile period of their menstrual cycle (2). However, most women often lack good knowledge as to when, during their menstrual cycle, they are most likely to get pregnant (3).

Maternal mortality is still high in Ethiopia, amounting to 412 per 100,000 live births (4). One of the most important factors in maternal mortality is unsafe abortion. Accordingly, women who do not have proper knowledge of the fertility period had gone to unsafe abortion (5). About half of all maternal deaths that occur in Africa resulted from unsafe abortion, which takes place mostly due to unintended pregnancy (6).

The knowledge of the fertility period is one of strategy for mitigating unintended pregnancies.

A study conducted by the World Health Organization (WHO) showed that two-thirds of women who wished to delay or limit childbearing stopped using modern contraception for fear of side effects, health concerns, and underestimation of the likelihood of conception (7).

A woman who knows her ovulatory period correctly will be safe from undesired and unplanned pregnancies. Similarly, non-users of contraception with no knowledge about their fertile period are at higher risk for unintended pregnancy (5). Moreover, couples who were actually practicing fertile period did not rely upon the notion of fertile period and most of them were not aware of their fertility period. Because of their limited knowledge, they go to reproductive healthcare services to seek help from healthcare providers (8). Furthermore, the knowledge of the fertile period has been one of the most contributory factors to the reduction of high fertility which have negative influences on economic and social development (9).

Providing adequate information on fertile times and related physiology would help women to understand the risk of pregnancy, plan their pregnancies appropriately, and recognize their pregnancies early (10).

Despite numerous advantages of usage of natural family planning methods, a study showed that some couples were challenged as it strains their interactions due to difficulties with abstinence, decreases in frequency and spontaneity, and unbalanced sexual drives between partners. Also, some of the couple's relationship were worsened as a result of anger, frustration, and misunderstandings resulting from partners' unmet sexual needs (11).

A study conducted in the United States showed that ~40% of women had no knowledge of the ovulatory period (12). A similar study conducted in the United States showed that 51% of women did not know the fertile period (13). Another study carried out in the USA among black women showed that nearly 91% of respondents had not correctly identified the time of the fertile period (14). A study in Australia found that about 40% of participants were unclear about when menstrual cycle design was most likely to occur (15). Likewise, a study on young people in Turkey found that knowledge about the fertility period was low, while only 40% of young people were well informed (16). Moreover, the results of a study conducted in Pakistan on certain adult population revealed that only 46% of them knew about the fertile period of the female cycle (17).

The study done among reproductive age women in 29 countries in sub-Saharan Africa using Demographic and Health Surveys (DHS) revealed that, only 8.3% had knowledge of ovulation which is generally low (18). A study done on Ghanaian male adolescents' knowledge about female fertile period showed that about 14.2% of them only correctly identified the specific fertile period within the female menstrual cycle (19). Similarly, a study conducted in Kenya found that less only than 8% of its respondents correctly identified their fertile years (20).

Findings from different studies indicate that some socio-demographic factors could affect women's knowledge about their fertile period. From these factors, sex, residence, economic status, and educational status were the commonest ones. Whereas, a study done in Turkey on University students' knowledge about fertile period showed that about half of female students, and one third of males were knowledgeable and correctly identified fertile period (16).

Demographic and Health Surveys conducted in 29 African countries among reproductive women showed that the knowledge about fertile period was low among young women, rural residents, women with poor economic status, and women with low levels of education (13, 18). A finding of a study done on Ghanaian male indicated that the knowledge about the female fertile period was significantly associated with the age of respondents, their level of education and region of residence (19).

Although there are numerous studies on the various natural methods of contraception, there are no studies that address the knowledge about the fertile period. Planning conception by having the knowledge of fertile period contributes a country such as Ethiopia with lowest level (1%) of natural family planning utilizers (21). Therefore, this study assessed the knowledge on fertile period and its determinants among reproductive age group women in Ethiopia.

## Methods

### Data Source, Sampling Technique, and Population

This study was based on the 2016 national cross-sectional study of Ethiopian demographic health survey (EDHS), the fourth survey conducted from January 18 to June 27, 2016, which used the Ethiopian Population and Housing Census (PHC) as a sampling frame. The frame was a complete list of 84,915 enumeration areas (EAs) in which each EA covers an average of 181 households.

The 2016 EDHS sample was stratified and selected in two stages. In the first stage, 645 EAs (202 in urban areas and 443 in rural areas) were selected. In the second stage, 28 households per cluster were selected from the newly created household listing. All necessary information about the sampling strategy, questionnaire, or other important information exists in the 2016 EDHS report (4). Finally, for this study, a total weighted sample of 15,683 reproductive age group women was involved.

### Variables of the Study

The outcome of this study was the knowledge about fertile period, which was classified into two categories: good knowledge and poor knowledge. The Knowledge of the ovulation period was assessed from the respondents by asking them a single question of “when do you think the ovulation period of a woman is?” Respondents who answered fertile period is at the middle of the menstrual cycle were categorized as with a good knowledge of fertile period while respondents who answered the question as fertile period is “during her period,” “after period ended,” “before period begins,” “at any time,” and “I don't know” were categorized as with a poor knowledge. The independent variables for this study were: age, residence, religion, ethnicity, partner's education, occupation, educational status, marital status, monthly income, media exposure, and internet utilization. These independent variables were developed using different literatures.

### Data Processing and Analysis

To make the data suitable for descriptive and analytical analyses, it was further coded using STATA version 14.0 software. In addition, sampling weights were done to adjust for non-proportional allocation of the sample to strata and regions during the survey process. Using a two-level binary logistic regression modeling, we examined the effect of a number of individual-level and community-level variables. Variables with *p*-value <0.05 were considered as significant predictors of knowledge on fertile period.

## Results

### Socio-Demographic Factors Characteristics of the Study Participants

From the total participants interviewed in EDHS, 15,683 weighted samples were involved in this study. Out of these study participants, 43.3% were Orthodox Christians. About 77.8% were living in rural areas, and 21.6% were in the age group of 15–19 years. The majority of the respondents and their husbands had no education, 47.8 and 45.8%, respectively. While half (49.9%) of them were unemployed. From the respondents, about 63.85% of them were married (Table 1).

**Table 1:** Socio demographic characteristic of the study participants in Ethiopia, 2016 (*N* = 15,683).

**Variables**	**Category**	**Weighted frequency (%)**
Age group	15–19 20–24 25–29 30–34 35–39 40–44 45–49	3,380 (21.56) 2,761 (17.61) 2,956 (18.85) 2,345 (14.95) 1,932 (12.32) 1,289 (8.22) 1,016 (6.48)
Residence	Urban Rural	3,476 (22.16) 12,207 (77.84)
Religion	Orthodox Protestant Muslin Others	6,786 (43.27) 3,674 (23.43) 4,892 (31.20) 330 (2.1)
Education	No education Primary Secondary Above secondary	7,498 (47.8) 5,490 (35) 1,817 (11.59) 877 (5.59)
Husbands/partners education	No education Primary Secondary Higher	4,763 (46.59) 3,772 (37) 975 (9.5) 713 (6.97)
Respondents occupation	Non-employee Employee	7,819 (49.86) 7,864 (50.14)
Husbands occupation	Non-employee Employee	807 (7.89) 9,416 (92.11)
Marital status	Never in union Married Widowed Divorced	4,036 (25.74) 10,223 (65.19) 429 (2.74) 994 (6.34)
Wealth index	Poorest Poorer Middle Richer Richest	2,633 (16.79) 2,809 (17.91) 2,978 (18.99) 3,099 (19.76) 4,163 (26.55)
Region	Tigray Amhara Oromia SNNPR Addis Ababa others	1,129 (7.2) 3,714 (23.68) 5,701 (36.35) 3,288 (20.97) 930 (5.93) 921 (5.87)

### Modifying Factors

The majority of the respondents (75.9%) had not heard of family planning on radio in the last few months, and from this, 79.6% of them had poor knowledge about fertile period. Likewise, from those respondents who haven't listened to the radio at all, 71.3% of them had poor knowledge. From those who haven't heard of family planning on TV in the last few months, about 86.4% of them had poor knowledge about fertile period. Out of the respondents, the majority (96.8%), who have never used internet, had poor knowledge about fertile period (Table 2).

**Table 2:** Modifying factors on knowledge about fertile period of respondents in Ethiopia, 2016 (*N* = 15,683).

**Variables**	**Category**	**Weighted frequency (%)**	**Poor knowledge**	**Good knowledge**
Heard family planning on radio last few months	No Yes	11,907 (75.93) 3,775 (24.07)	9,540 (79.60) 2,445 (20.40)	2,368 (64.02) 1,330 (35.98)
Frequency of listening to radio	Not at all Less than once a week	10,338 (65.92) 2,644 (16.86)	8,546 (71.31) 1,835 (15.31)	1,939 (52.43) 782 (21.13)
	At least once a week	2,701 (17.22)	1,603 (13.38)	978 (26.43)
Heard family planning on tv last few months	No Yes	12,844 (81.90) 2,838 (18.10)	10,349 (86.35) 1,635 (13.65)	2,495 (67.48) 1,203 (32.52)
Use of internet	Never Yes, last 12 months Yes, before last 12 months	14,903 (95.03) 693 (4.42) 86 (0.55)	11,601 (96.80) 328 (2.74) 55 (0.46)	3,303 (89.30) 364 (9.87) 30 (0.83)

### Factors Associated With Knowledge About Fertile Period

From the respondents, 23.6% (95% *CI*: 23–24) of them had good knowledge about fertile period. Age from 20 to 49 years, protestant religion, respondent's level of education, partner's level of education, wealth index, heard of family planning, listening to the radio, frequency of watching TV, and internet utilization were significant individual factors while exposure to community FP messages was the significant factor from community level factors.

The odds of good knowledge were 1.51 times higher in the age group of 20–24 years (*AOR* = 1.51; *CI* = 1.15–1.97). Similarly, the odds were 1.63 times higher in the age group of 25–29 years (*AOR* = 1.63; *CI* = 1.25–2.13); 1.99 times higher in the age group ranging from 30 to 34 years (*AOR* = 1.91; *CI* = 1.45–2.52); 1.51 times higher in the age group of 35–39 years (*AOR* = 1.51; *CI* = 1.14–2.01); 1.74 times higher in the age group of 40–44 years (*AOR* = 1.74; *CI* = 1.27–2.35); and 1.55 times higher in the age group of 45–49 years (*AOR* = 1.55; *CI* = 1.11–2.15) than the age group of 15–19 years.

Accomplishment of primary education increased the odds of good knowledge by 1.35 times than it did with those with no education (*AOR* = 1.35; *CI* = 1.16–1.57), while it is 2.03 times with those who completed secondary education (*AOR* = 2.03; *CI* = 1.61–2.55) and 2.61 times (*AOR* = 2.61; *CI* = 1.94–3.53) more with those who completed higher education as compared to those with no education at all. Similarly, high level of partner's education attainment (*AOR* = 1.36; *CI* = 1.10–1.73) increased the odds of knowledge about fertile period by 1.36 times as compared to respondents with no formal education.

Wealth index of poorer (*AOR* = 1.34; *CI* = 1.09–1.65), middle (*AOR* = 1.29; *CI* = 1.03–1.61), richer (*AOR* = 1.41; *CI* = 1.11–1.78), and richest (*AOR* = 1.62; *CI* = 1.20–2.17) increased the odds of knowledge about fertile period than the poorest group did.

A person listened a radio < once a week has increased the odds of good knowledge by 1.2 times (*AOR* = 1.2; *CI* = 1.02–1.42); similarly, watching TV at least once a week has increased the odds of good knowledge by 1.4 times (*AOR* = 1.4; *CI* = 1.11–1.75) than with those who never watched TV at all. Whereas, the odds of good knowledge is increased by 1.3 times among those who ever heard of family planning (*AOR* = 1.3; *CI* = 1.12–1.52). Furthermore, internet utilization in the last 12 months (*AOR*: 1.55 (1.17, 2.05) increased the odds of knowledge about fertile period by 1.55 times among users as compared to those who never used internet.

Among community level variables, high exposure to community FP messages increased the odds of knowledge about fertile period by 1.52 times than low community FP messages exposure did (*AOR* = 1.52; *CI* = 1.16–2.0) (Table 3).

**Table 3:** A multivariable multilevel analysis of Individual and community level factors associated with knowledge of fertile period among reproductive age women in Ethiopia, 2016 (*N* = 15,683).

**Variables**	**Model I**	**Model II**	**Model III**	**Model IV**
		**AOR (95% CI)**	**AOR (95% CI)**	**AOR (95% CI)**
**Age**	–		–	
15–19	–	1	–	1.00
20–24	–	1.52 (1.16, 1.98)^*^	–	1.51 (1.15, 1.97)^*^
25–29	–	1.66 (1.27, 2.16)^*^	–	1.63 (1.25, 2.13)^*^
30–34	–	1.94 (1.48, 2.56)^*^	–	1.91 (1.45, 2.52)^*^
35–39	–	1.55 (1.16, 2.10)^*^	–	1.51 (1.14, 2.01)^*^
40–44	–	1.78 (1.32, 2.40)^*^	–	1.74 (1.27, 2.35)^*^
45–49	–	1.58 (1.14, 2.19)^*^	–	1.55 (1.11, 2.15)^*^
**Religion**	–		–	
Orthodox	–	1	–	1.00
Catholic	–	1.09 (0.53, 2.23)	–	1.05 (0.51, 2.15)
Protestant	–	0.85	–	0.81 (0.65, 0.99)^*^
Muslim	–	1.03 (0.88, 1.22)	–	0.99 (0.83, 1.18)
Traditional	–	0.71 (0.29, 1.78)	–	0.69 (0.28, 1.70)
Other	–	0.64 (0.27, 1.52)	–	0.59 (0.25, 1.41)
**Respondent education**				
No formal education	–	1	–	1.00
Primary	–	1.36 (1.17, 1.59)^*^	–	1.35 (1.16, 1.57)^*^
Secondary	–	2.04 (1.63, 2.57)^*^	–	2.03 (1.61, 2.55)^*^
Higher	–	2.67 (2.00, 3.60)^*^	–	2.61 (1.94, 3.53)^*^
**Partner's education**				
No formal education	–	1	–	1.00
Primary	–	1.12 (0.96, 1.30)	–	1.10 (0.95, 1.28)
Secondary	–	1.13 (0.92, 1.40)	–	1.11 (0.90, 1.37)
Higher	–	1.39 (1.10, 1.76)^*^	–	1.36 (1.10, 1.73)^*^
Don't know	–	1.06 (0.59, 1.89)	–	1.05 (0.58, 1.87)
**Currently working**				
No	–	1	–	1.00
Yes	–	1.05 (0.93, 1.19)	–	1.04 (0.92, 1.18)
**Wealth index**				
Poorest	–	1	–	1.00
Poorer	–	1.37 (1.11, 1.68)^*^	–	1.34 (1.09, 1.65)^*^
Middle	–	1.34 (1.08, 1.66)^*^	–	1.29 (1.03, 1.61)^*^
Richer	–	1.51 (1.20, 1.89)^*^	–	1.41 (1.11, 1.78)^*^
Richest	–	2.02 (1.59, 2.57)^*^	–	1.62 (1.20, 2.17)^*^
**Heard of FP**				
No	–	1	–	1.00
Yes	–	1.32 (1.13, 1.53)^*^	–	1.30 (1.12, 1.52)^*^
**Frequency of listening to radio**				
Never	–	1	–	1.00
Less than once a week	–	1.20 (1.02, 1.42)^*^	–	1.20 (1.02, 1.42)^*^
At least once a week	–	1.06 (0.89, 1.27)	–	1.07 (0.89, 1.27)
**Frequency of watching TV**	–		–	
Never	–	1	–	1.00
Less than once a week	–	1.15 (0.93, 1.41)	–	1.12 (0.91, 1.37)
At least once a week	–	1.47 (1.17, 1.84)^*^	–	1.40 (1.11, 1.75)^*^
**Heard of FP from TV**	–		–	
No	–	1	–	1.00
Yes	–	1.15 (0.94, 1.42)	–	1.12 (0.91, 1.38)
**Internet use**				
Never		1	–	1.00
Yes, last 12 months	–	1.56 (1.17, 2.07)^*^	–	1.55 (1.17, 2.05)^*^
Yes, before last 12 months	–	2.05 (0.97, 4.36)	–	1.99 (0.94, 4.26)
**Residence**	–	–		
Rural	–	–	1	1.00
urban	–	–	1.76 (1.37, 2.27)^*^	1.30 (0.94, 1.80)
**Community poverty**	–	–		
High	–	–	1	1.00
Low	–	–	1.32 (1.06, 1.65)^*^	1.13 (0.87, 1.47)
**Contextual region**	–	–		
Metropolitans	–	–	1	1.00
Agrarian	–	–	0.90 (0.76, 1.07)	0.82 (0.67, 1.02)
Pastoralist	–	–	0.63 (0.46, 0.86)^*^	0.80 (0.56, 1.15)
**Big community health facility distance problem**	–	–		
Low	–	–	1	1.00
High	–	–	1.04 (0.87, 1.25)	1.19 (0.96, 1.46)
**Community media exposure**	–	–		
Low	–	–	1	1.00
High	–	–	1.06 (0.85, 1.33)	0.99 (0.77, 1.28)
**Community ANC utilization rate**	–	–		
Low	–	–	1	1.00
High	–	–	1.06 (0.86, 1.29)	0.97 (0.78, 1.22)
**Community female education**	–	–		
Low	–	–	1	1.00
High	–	–	1.13 (0.92, 1.40)	0.86 (0.67, 1.09)
**Community FP messages exposure**	–	–		
Low	–	–	1	1.00
High	–	–	1.85 (1.45, 2.34)^*^	1.52 (1.16, 2.0)^*^
**Community FP utilization rate**				
Low	–	–	1	1
High	–	–	0.91 (0.77, 1.06)	0.85 (0.70, 1.02)

* *p-value < 0.05*.

## Discussion

The aim of this study was to determine the knowledge about the fertile period and its determinant factors among women in reproductive age groups in Ethiopia, using the national 2016 EDHS data. Among the determinant factors; age from 20 to 49 years, protestant religion, respondent's level of education, partner's level of education, wealth index, heard of family planning, listening to the radio, frequency of watching TV, and internet utilization were significant factors from individual factors while exposure to community FP messages was one of the significant factors from community-level factors, which were found be significantly associated with the knowledge about fertile period.

Those who knew about the fertile period in this study were about 23.6% (95% *CI*: 23–24). This finding is higher than the findings of DHS studies done in Ghana and Kenya (18–20). This might be attributed to the differences that exist in religious and cultural practices. But, it is lower than studies done in Turkey, USA, and Australia, and this could be due to better health education access and literacy rate in these developed countries (10, 15, 16). Moreover, a study indicated that poor communication about sexual matters in the family, school, and community was a reason to women in Africa not to have a better knowledge about the ovulatory period (18).

The current study indicated that having good knowledge was higher in the age group of 20–24 years than the reference group, which is used in contrast with this study and done in USA, which revealed that the age group of 18–24 years demonstrated less knowledge regarding ovulation period (12) and DHS done in some African countries showed that young women who are in the age group of 20–24 years are less likely to have good knowledge of ovulation timing (18). This might be due to socioeconomic and demographic differences among the nations.

The Protestant religion was found to decrease the odds of knowledge about fertile period than Orthodox Christian religion did. The possible explanation may be due to the difference in religious teachings of the two churches on modern contraception. In protestant rituals, it is allowed for women to use modern contraception; thus, they do not depend and need to know about their fertile period unlike that of the Orthodox Christian religion.

The finding of this study showed that those respondents at the richest wealth status had good knowledge than those groups with the poorest wealth index. This is similar to the result of DHS study done in 29 countries (13, 18). Moreover, in this study, respondents with higher level education were found to have good knowledge than with those respondents with low-level. This is in line with the DHS finding which revealed that knowledge about fertile period was low among respondents with low levels of education (18). Also, the partner's level of education was found to positively affect knowledge about ovulation. This finding is supported by a study done in Ghana, in which a higher level of education of males contributed to the good knowledge about ovulation (19). This could be due to the possibility of delivering education about the female fertile period is higher at a higher level of education.

Those respondents who listened to the radio and watched TV were found to have good knowledge than those who never listened to the radio at all and who never watched TV. The possible explanation for this finding is that these groups are more likely to be exposed to the essential health information because there are a lot of programs held on contraception and related health issues in such Medias. In addition, whoever heard about family planning was found to have good knowledge about fertile period. From the community level factors of the current study, community FP messages exposure increased the likelihood of having a good level of knowledge about the fertile period. Thus, the likelihood of having good knowledge on the ovulatory period is higher among the ones that were exposed than with those who did not get a chance to be exposed to such Medias and FP messages.

The limitation of this study is, as the present study was extracted from EDHS data which were collected for another purpose, that it has limited us from incorporating other important determinants of knowledge about fertile period. However, with the above limitation, the present study tried to picture out the national magnitude of knowledge about fertile period and its determinants among reproductive age women of the country.

## Conclusion

The number of reproductive-age women who know about the fertile period is low in Ethiopia. Age above 19 years, respondent's education attainment from primary to higher education, partner high level of education, being from poorer to richest wealth status, listening radio, watching TV, ever heard of FP, internet usage in the last 12 months, being protestant religion followers, and community family planning exposure were significantly associated with good knowledge about fertile period. Hence, awareness-raising about the female fertile period for Ethiopian women of reproductive age is required with due emphasis to partner with lower level of education, illiterate respondent, being protestant religion followers, internet non-users, and to those who do not watch TV, who do not listen to radio, and who do not have exposure to community family planning messages.

## Data Availability Statement

The data analyzed in this study is subject to the following licenses/restrictions: The datasets supporting the conclusions of this article are available upon request to the corresponding author. Requests to access these datasets should be directed to ZA, znigussie35@gmail.com.

## Ethics Statement

The study used data from the 2016 EDHS. Since it is secondary data from demographic and health survey, we accessed the data set based upon request (www.dhsprogram.com online) and there was no ethical approval required.

## Author Contributions

MW conceptualized the study, analyzed the data, and drafted the manuscript. AK, KS, and ZA participated in design of the study and critically revised the manuscript. All authors read and approved the final manuscript.

## Conflict of Interest

The authors declare that the research was conducted in the absence of any commercial or financial relationships that could be construed as a potential conflict of interest.

## Publisher's Note

All claims expressed in this article are solely those of the authors and do not necessarily represent those of their affiliated organizations, or those of the publisher, the editors and the reviewers. Any product that may be evaluated in this article, or claim that may be made by its manufacturer, is not guaranteed or endorsed by the publisher.

## Abbreviations

EA: Enumeration areas
EDHS: Ethiopian Demographic and Health Surveys
FP: Family planning
PHC: Population and Housing Census
SNNPR: Southern Nations, Nationalities and People's Region
USA: United States of America
WHO: World Health Organization.
